# Antimicrobial Potential of Metabolites in Fungal Strains Isolated from a Polluted Stream: *Annulohypoxylon stygium* WL1B5 Produces Metabolites against Extended-Spectrum Beta-Lactamase-Positive *Escherichia coli*

**DOI:** 10.3390/antibiotics12010027

**Published:** 2022-12-24

**Authors:** Walter Oliva Pinto Filho Segundo, Roberta Silva de Oliveira, Rildo Mendes Lima, Paulo Alexandre Lima Santiago, Luciana Aires de Oliveira, Ana Cláudia Alves Cortez, Emerson Silva Lima, Érica Simplício de Souza, Hagen Frickmann, João Vicente Braga de Souza

**Affiliations:** 1 Program in Biodiversity and Biotechnology of the Bionorte Network (PPG-BIONORTE), Amazonas State University (UEA), Manaus 69065-001, AM, Brazil; 2 Mycology Laboratory, National Institute for Amazonian Research (INPA), Manaus 69067-375, AM, Brazil; 3 Central Public Health Laboratory of the State of Amazonas (LACEN/AM), Manaus 69020-040, AM, Brazil; 4 Department of Biology, Amazonas State University (UEA), Tabatinga 69640-000, AM, Brazil; 5 Faculty of Pharmaceutical Sciences, Federal University of Amazonas (UFAM), Manaus 69067-005, AM, Brazil; 6 Higher School of Technology, Amazonas State University (UEA), Manaus 69050-020, AM, Brazil; 7 Department of Medical Microbiology, Virology and Hygiene, University Medicine Rostock, 18057 Rostock, Germany; 8 Department of Microbiology and Hospital Hygiene, Bundeswehr Hospital Hamburg, 20359 Hamburg, Germany

**Keywords:** fungi, antimicrobial, bacteria, resistance, *Escherichia coli*

## Abstract

The emergence of multidrug resistance in bacterial pathogens is a growing public health concern requiring solutions including the discovery of new antimicrobial drugs. Fungi have been used for decades as a source of antimicrobials. Ongoing screenings for newly characterized fungal strains producing antimicrobials include environments that are difficult to access like the deep sea, glaciers, wastewaters and environments polluted due to human activity. In the present study, fungal microorganisms were isolated from water samples taken from a polluted stream in the city of Manaus, AM, Brazil, and screened for antimicrobial effects against *Escherichia coli*. Using extracts from five isolates (*Annulohypoxylon stygium* WL1B5, *Colletotrichum fructicola* WL3B9, *Clonostachys rosea* WL5B18, *Clonostachys rosea* WL8B28 and *Trichoderma harzianum* WL9B49), antimicrobial activity against the reference strains *Escherichia coli* ATCC 25922 as well as *E. coli* NCTC 13353, an extended-spectrum beta-lactamase-positive strain, was observed. Inhibition zones ranged from 1 to 35.9 mm and a minimum inhibitory concentration of 400 µg/mL could be demonstrated. Assessments of the metabolites of *Annulohypoxylon stygium* WL1B5 allowed us to identify nodulisporone and daidzein, which have already been associated with antimicrobial activity. The findings confirm the feasibility of isolating fungal strains from polluted sites producing metabolites that can serve as potential future alternatives for the treatment of multidrug-resistant bacteria.

## 1. Introduction

The international emergence of multidrug-resistant or even pan-drug-resistant bacterial pathogens is an ongoing concern of global healthcare and public health. The World Health Organization (WHO) predicts that by 2050, multidrug-resistant bacteria will likely be causally associated with the death of approximately 10 million people per year [1].

Starting with the clinical application of antibiotic drugs (e.g., penicillins and cephalosporines) several decades ago, evolutionary selection in many bacterial species has driven the spread of various distinct mechanisms of resistance to several classes of antibiotics used for therapeutic purposes in both outpatient medical facilities and hospitals [2]. Bacterial pathogens such as methicillin-resistant *Staphylococcus aureus* (MRSA), vancomycin-resistant *Enterococcus* spp. (VRE), penicillin-resistant *Streptococcus pneumoniae* and extended-spectrum beta-lactamase (ESBL)-producing Enterobacterales have emerged, posing a challenge to antimicrobial therapies [3].

Filamentous fungi from the phylum Ascomycota are known to produce metabolites with antimicrobial activity such as alkaloids, terpenoids, polyketides, polysaccharides, steroids, quinones, flavonoids, aliphatic compounds and phenols [4]. As recently summarized, most new, naturally occurring substances with antimicrobial activity described in the literature have been isolated from fungi; several such compounds have facilitated the development of new, clinically important antimicrobial agents [5]. As bacterial resistance is a constant concern, there is an ongoing need for new antimicrobial substances with sufficient biological activity and low therapeutic side effects allowing their use for medical therapies.

Severely polluted streams have been shown to harbor a high number of fungal species with the potential of producing bioactive metabolites [6,7]. Associated with the strong selection pressure on local microbial sentinel communities in such toxic environments, fungi isolated from such locations have been shown to produce more potent secondary metabolites than those that are commonly produced under stabler conditions. Therefore, such environments are likely to host a wide variety of fungal strains with promising antimicrobial bioactivity. As a proof-of-principle assessment, the objective of this work was to investigate the presence of cultivable filamentous fungi in a severely polluted stream in Manaus City, Amazonas, Brazil, and their ability to produce substances with antimicrobial activity.

## 2. Results

### 2.1. Collection Sites

**Figure A1:**
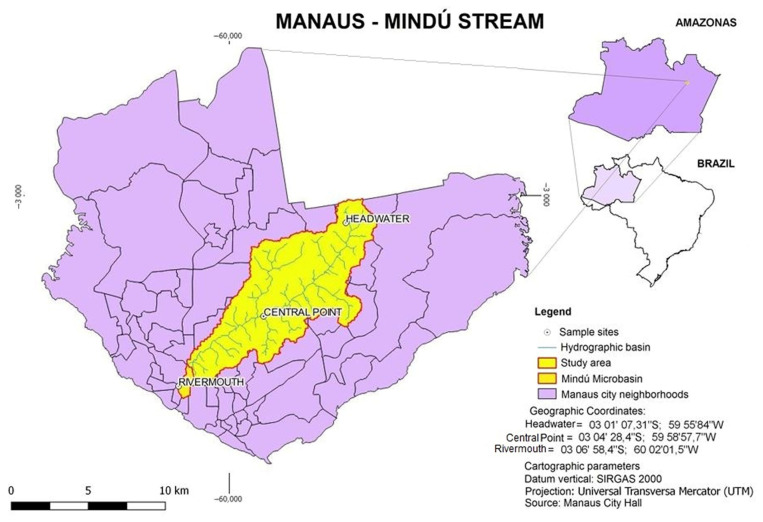
Map of Manaus city (Amazonas, Brazil) showing the research site and sampling points distributed at Mindú stream.

Physical and chemical analyses were carried out with water taken at various sampling sites on the Mindú stream (Table 1, Appendix A Figure A1). The data indicate that the sampling points “central point” and “estuary” were particularly impacted by pollution due to industrial or domestic waste being dumped into the water. Even though the temperature and pH of the water remained similar, the changes of other variables such as dissolved oxygen, turbidity, electrical conductivity and total nitrogen indicated inconsistent water quality between the different sampling sites.

### 2.2. Fungal Isolates

**Figure 1:**
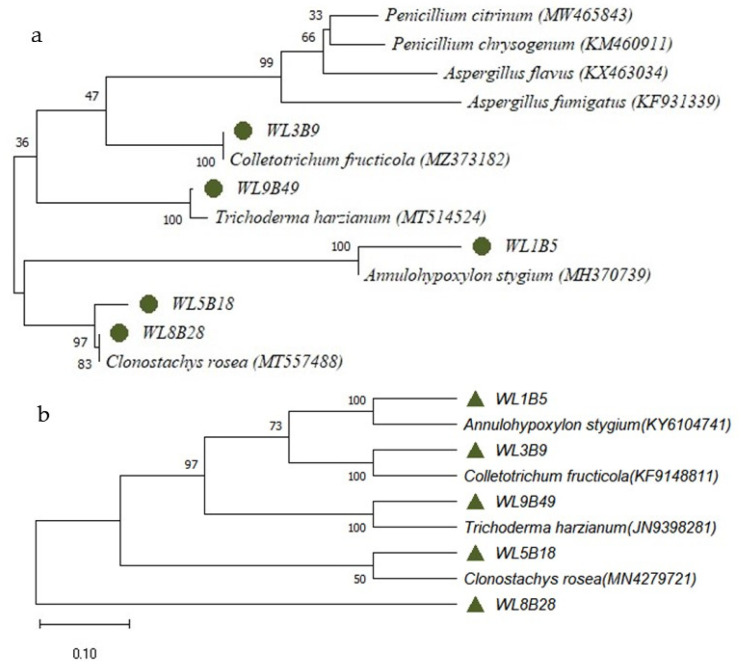
Phylogenetic tree constructed based on the sequences of ITS1-5.8S rRNA gene-ITS2 (**a**) and the D1/D2 domain of the 28S rRNA gene (**b**) using the maximum likelihood method and the Tamura-Nei model [8]. The percentage of trees in which the associated taxa clustered together in the bootstrap test (1000 replicates) is shown next to the branches. The tree is drawn to scale, with branch lengths indicating the number of substitutions per site. This analysis involved 22 nucleotide sequences. The green dots/pyramids indicate the isolates from this study. The others are previously identified sequences of antimicrobial substance-producing fungi. There were a total of 1318 positions in the final dataset. Evolutionary analyses were conducted by applying MEGA X v10.2.4.

A total of 67 fungal isolates were obtained from the sampling sites. Conventional identification based on morphological assessments allowed the taxonomic assignment of the genera of 21 isolates (Table 2). In addition, 5 isolates that were identified as antimicrobial substance producers in the present work were further differentiated at the species level—using DNA sequencing based on ITS1-5.8S rRNA gene-ITS2 and 26 rRNA gene fragments—as *Annulohypoxylon stygium* (sample ID WL1B5), *Colletotrichum fructicola* (samples ID WL3B9), *Clonostachys rosea* (sample ID WL5B18) (syn. *Gliocladium roseum*), *Clonostachys rosea* (sample ID WL8B28) and *Trichoderma harzianum* (sample ID WL9B49) (Figure 1). The nucleotide sequences obtained after amplification and sequencing were deposited at NCBI GenBank with the accession numbers OP580168, OP578038, OP563025, OP578133 and OP578134, respectively.

### 2.3. Production of Antibacterial Substances by the Fungal Isolates

In order to investigate the potential production of substances with antibacterial activity by the fungal isolates, all 67 isolates were subjected to submerged bioprocessing for 14 days. The fungal cultures were extracted applying a hexane- and EtOAc-based procedure, as detailed in the methods chapter. The obtained fractions were tested by agar diffusion testing for antimicrobial activity against *E. coli* ATCC 25922 and the extended-spectrum beta-lactamase- (EBSL-)producing strain *E. coli* NCTC 13353. Table 3 shows the antibacterial activity of hexane-based, EtOAc-based and crude extracts with inhibition zone diameters ranging from 1 to 35.9 mm. Among the 67 fungal isolates investigated, metabolites of 5 isolates were associated with halos of bacterial growth inhibition. Metabolites from the isolates *A. stygium* (WL1B5), *C. rosea* (WL5B18) and *C. rosea* (WL8B28) were found to induce the largest inhibition halos when tested with *E. coli* ATCC 25922 and the ESBL-positive *E. coli* strain NCTC 13353.

#### Minimum Inhibitory Concentration—MIC

The minimal inhibitory concentrations of EtOAc extracts of *A. stygium* WL1B5, *C. rosea* WL5B18 and *C. rosea* WL8B28 were assessed by applying a microdilution assay with *E. coli* ATCC 25922 and the ESBL-positive strain *E. coli* NCTC 13353. The EtOAc extracts of these three fungal isolates yielded a MIC of 400 µg/mL for both assessed bacterial strains (Table 4).

### 2.4. In Vitro Cytotoxicity Assay

In order to study the toxicity of the fungal metabolites, the cytotoxicity, i.e., lethal effect on cell viability, of the metabolites of EtOAc extracts of *A. stygium* WL1B5, *C. rosea* WL5B18 and *C. rosea* WL8B28 was assessed with MRC5 human lung fibroblast cells. The EtOAc extracts of *A. stygium* WL1B5, *C. rosea* WL5B18 and *C. rosea* WL8B28 showed an IC50% > 100 µg/mL, indicating low toxicity to human cells of the MRC5 line. Doxorubicin, which was used as an experimental positive control, showed a much lower IC50% of 5 µg/mL.

### 2.5. TLC and Contact Bioautography

**Figure A2:**
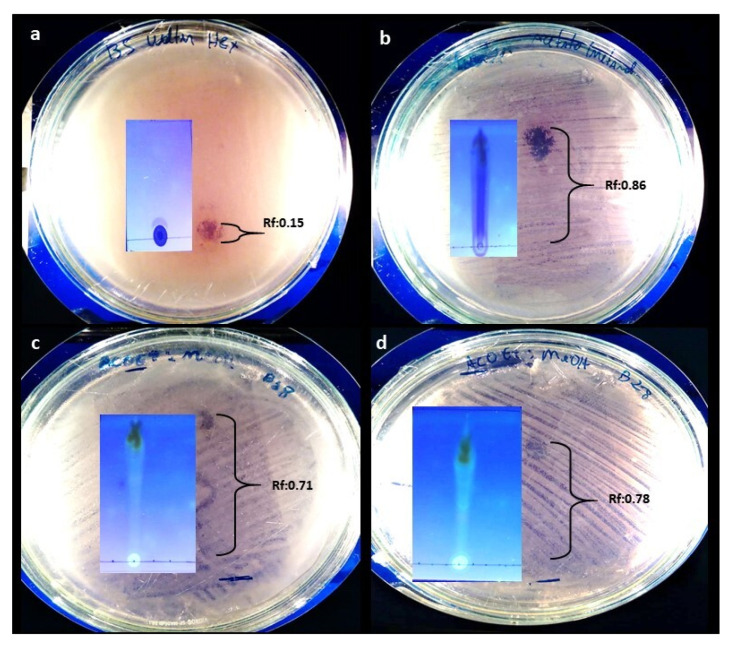
Mobile phase, main bands and the Rf value of the bands that were associated with antibacterial activity against *E. coli* ATCC 25922. *A. stygium* WL1B5 – Hex 100% (**a**), MeOH 100% (**b**); *C. rosea* WL5B18—EtOAc/MeOH 1:1 *v/v* (**c**); *C. rosea* WL8B28—EtOAc/MeOH 1:1 *v/v* (**d**).

TLC assays for the analysis of EtOAc extracts were carried out with *A. stygium* WL1B5, *C. rosea* WL5B18 and *C. rosea* WL8B28 using the mobile phases hexane (100%), hexane/ethyl acetate (1:1, *v*/*v*), ethyl acetate (100%), ethyl acetate/methanol (1:1, *v*/*v*) and methanol (100%). In addition, a bioautography assay was conducted with the TLC platelets to identify the chromatographic bands with antibacterial activity. Table 5 presents the mobile phase, main bands and the Rf value of the bands that were associated with antibacterial activity against *E. coli* ATCC 25922 (Appendix A Figure A2).

### 2.6. LC-MS/MS Analysis

**Figure 2:**
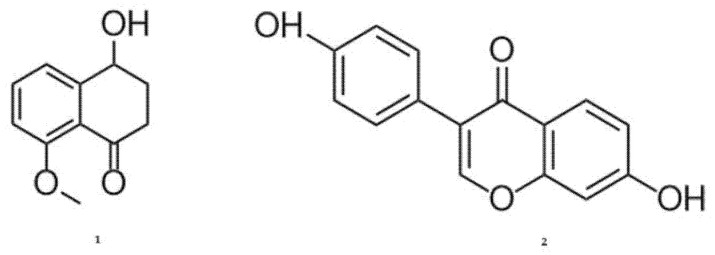
Structures of nodulisporone sodium (**1**) and daidzein (**2**) detected by LC-MS/MS in the EtOAc extract of the fungus *A. stygium* WL1B5. Chemical structures were drawn using the ChemDraw Professional 18.1 software (PerkinElmer Informatics, Waltham, Massachusetts, United States).

**Figure A3:**
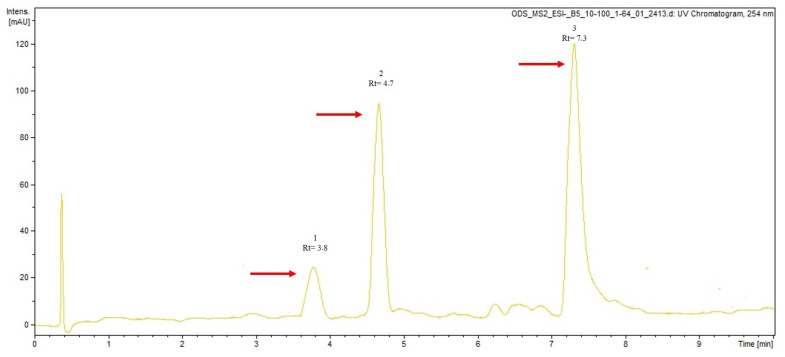
Chromatograms (UV 254 nm) of EtOAc extract of *A. stygium* WL1B5 by LC-MS/MS and the three major peaks at 3.8 (C1), 4.7 (C2) and 7.3 (C3) min. During the research in the literature for information on the peak eluting at 4.7, no equivalent mass compounds were found.

**Figure A4:**
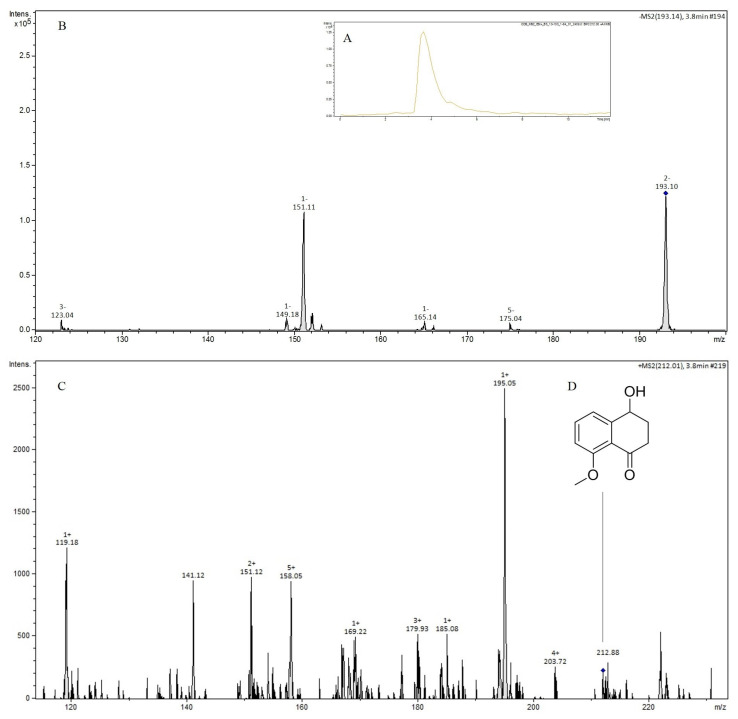
(**A**) Peak of the MS2 spectrum; (**B**) MS2 spectrum of [M − H] *m*/*z* 193 in negative mode; and (**C**) [M + H] *m*/*z* 212 in positive mode of compound 1 from LC-MS/MS analysis of EtOAc extract produced by *A. stygium* WL1B5; (**D**) Nodulisporone. The assessment was performed without replicates.

**Figure A5:**
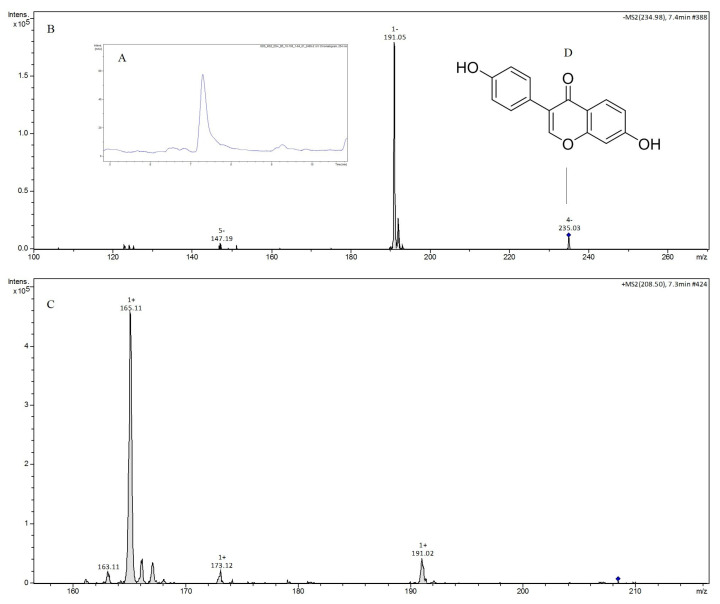
(**A**) Peak of the MS2 spectrum; (**B**) MS2 spectrum of [M − H] *m*/*z* 234 in negative mode; (**C**) [M + H] *m*/*z* 208 in positive mode of compound 3 from LC-MS/MS analysis of EtOAc extract produced by *A. stygium* WL1B5. (**D**) Daidzein.

When the EtOAc extract of *A. stygium* WL1B5 was further investigated using LC-MS/MS, the chromatograms showed three major peaks at 3.8, 4.7 and 7.3 min (Appendix A Figure A3). The peak eluting at 3.8 min had the main ion compounds of [M-H] *m/z* 193 and [M + H] *m/z* 212, the peak eluting at 4.7 had no ion compounds equivalent to the mass found and the peak eluting at 7.3 min had the main ion compounds of [M-H] *m*/*z* 234 and [M + H] *m/z* 208 (Appendix A Figure A4 and Figure A5). Figure 2 presents likely metabolites abundant in the EtOAc extract of *A. stygium* WL1B5. Two substances were suggested by the LC-MS/MS results: nodulisporone sodium (**1**) and daidzein (**2**).

## 3. Discussion

The findings of this study confirm that isolated fungal strains from polluted environments like streams show the potential to produce antimicrobially active substances. In summary, *A. stygium* WL1B5, *C. fructicola* WL3B9, *C. rosea* WL5B18, *C. rosea* WL8B28 and *T*. *harzianum* WL9B49 were able to synthesize metabolites with activity against *E. coli* ATCC 25922 and the ESBL-positive *E. coli* strain NCTC 13353 when grown under submerged fermentation conditions. Applying EtOAc extraction, the most pronounced antimicrobial activity could be demonstrated. With the help of LC-MS/MS analysis, two active metabolites were proposed in the EtOAc extract of the *A. stygium* WL1B5 isolate.

The assessed water samples indicated an increase in pollution between the sampling sites “head waters” and “estuary”, suggesting a continuous load of domestic/industrial waste which is dumped in the Mindú stream (Appendix A Figure A1) [9]. Concomitantly, there was an increase in the number of isolated fungi. Most of the fungi were plant-associated, and a relevant proportion could not be identified due to the absence of reproductive elements. This result was expected, because in addition to domestic sewage, the Mindú stream also harbors riparian vegetation, which is characteristic of the Amazon region.

Our findings further demonstrated that the isolates *Annulohypoxylon stygium* WL1B5, *Colletotrichum fructicola* WL3B9, *Clonostachys rosea* WL5B18, *Clonostachys rosea* WL8B28 and *Trichoderma harzianum* WL9B49 produced metabolites with antibacterial activity. Specifically, metabolites in the EtOAc extracts of *Annulohypoxylon stygium* WL1B5, *Clonostachys rosea* WL5B18, *Clonostachys rosea* WL8B28 could be associated with activity against an *E. coli* reference strain with susceptibility towards third-generation cephalosporines and the ESBL-positive strain *E. coli* NCTC 13353. This finding matches with previous works that demonstrated activity of metabolites of *A. stygium* against *Bacillus subtilis*, *E. coli* ATCC 25922, methicillin-resistant *S. aureus* (MRSA), *S*. *aureus* ATCC 25923 and *Candida albicans* [10,11,12,13]. The results are also in line with previous works carried out with *C. rosea* species, which had suggested antimicrobial activity of fungal metabolites against *E. coli*, *S. aureus*, *Bacillus cereus* and *Citrobacter freundii* [14,15,16,17].

Of note, the AcOEt extracts of *A. stygium* WL1B5, *C. rosea* WL5B18 and *C. rosea* WL8B28 were shown to be associated with only a minor cytotoxic effect on MRC5 cells. This finding is in agreement with previously published results on the comparatively low toxicity of *A. stygium* metabolites [17,18]. Similar results have also been reported for fungal metabolites produced by *Clonostachys rosea* [19,20].

HLPC-MS/MS analysis of the EtOAc extract of *A. stygium* WL1B5 proposed the abundance of the metabolites nodulisporone and daidzein. Nodulisporone is a tetralone originally isolated from fungi of the *Nodulisporium* genus (Ascomycota, *Xylariacea*) [21] and recently also demonstrated in fungi of the *Annulohypoxylon* genus [22]. In EtOAc extracts of *Xylaria* polymorpha (*Xilariaceae*), this compound has previously shown antimicrobial inhibition zones with *Candida albicans* strains (10 mm) and *T. harzianum* strains [13 mm) [22]. Tetralones have been described to interfere with DNA replication by inhibiting the cellular DNA topoisomerase, thus showing effects on gene expression and recombination of microorganisms [23].

Daidzein is a well-characterized flavonoid produced by plants with several pharmacological properties. This metabolite has already been identified in the genera *Aspergillus*, *Penicillium* and *Trichoderma* [24,25,26]. However, this is the first time that daidzein is reported in an *Annuloxypoxylon* species. When previously isolated from *Trichoderma* sp. YM 311505, this compound had exhibited antibacterial activity against *E. coli* with a minimum inhibitory concentration (MIC) of 64 µg/mL [26]. As a component of of EtOAc extracts of *Aspergillus tubingensis*, antimicrobial activity against several strains of *Vibrio* species with MIC values between 0.5 µg/mL to 1.0 μg/mL have been associated with this substance [27]. Flavonoids are known for their inhibiting effects on bacterial nucleic acid synthesis and metabolism as well as for effects on the cellular membrane permeability [28].

The present work opens perspectives for future studies focusing on a) a deeper identification of bioactive molecules responsible for the observed antibacterial effects; b) test assays for antimicrobial effects including Gram-positive strains and c) elucidation of the substances applying nuclear magnetic resonance (NMR) analyses. Hence, the here presented data can serve as a basis for ongoing studies, contributing to a better understanding of the chemical and biological characteristics of the isolates. The discovery of new bioactive substances such as natural antibiotics as important resources to counteract the problem of increasing drug resistance in Gram-negative bacterial pathogens like ESBL-positive *E. coli* will remain a relevant focus for future research activities.

## 4. Materials and Methods

### 4.1. Sample Collection

Five water samples were collected from the polluted stream Mindú at three sampling sites in Manaus city, Amazonas, Brazil. In the following, these sampling sites are addressed as “head water” (coordinates 03 01′07.31″ S 59 55′84″ W), “central point” (coordinates 03 04′28.4″ S 59 58′57.7″ W) and “estuary” coordinates (03 06′58.4″ S 60 02′01.5″ W). In line with the recommendations by the American Public Health Association Standards [29], all samples were taken from a depth of 15 to 30 cm, kept in sterile flasks, stored in an icebox at 4–8 °C and transported to the mycology laboratory of the National Institute of Amazonian Research for the isolation of fungi. Physical and chemical water parameters were measured on site using a Profline 197i Multiparameter Water Quality device (WTW, Wellheim, Germany).

### 4.2. Isolation of Fungi from the Polluted Water

Next, 1000 µL of sample material was inoculated via the spread plate technique on laboratory-produced Sabouraud agar (peptone 10 g/L, dextrose 20 g/L, agar 20 g/L) (Kasvi, Madrid, Spain) plates, to which the antibiotic drug chloramphenicol (250 mg/L) was added. Individual fungal strains were isolated by plating onto fresh Potato Dextrose Agar (PDA, Kasvi, Madrid, Spain) and incubating at 28 °C for 7 days. Each strain was identified to the genus level by assessing the morphological characteristics based on the microscopic description of conidia, phialides, metule, conidiophore and hyphae.

### 4.3. Molecular Fungal Identification

The selected fungi were identified based on both morphological and ITS (internal transcribed spacer) as well as 28S rRNA gene sequence analysis. Genomic DNA was isolated according to a previously described protocol [30]. The ITS sequence of the fungal isolate was amplified using the forward primer ITS1 (5′ CTTGGTCATTTAGAGGAAGTAA-3′) and the reverse primer ITS4 (5′ CTTGGTCATTTAGAGGAAGTAA-3′) as described elsewhere [31]. The D1/D2 domain of the 28S rRNA gene was amplified applying the primer pair NL1 (5′-GCATATCAATAAGCGGAGGAAAAG-3′) and NL4 (5′-GGTCCGTGTTTCAAGACGG-3′) using a published protocol [32]. Sequences of the fungal ITS and rRNA gene regions were compared with those deposited in the NCBI (National Center for Biotechnology Information; http://www.ncbi.nlm.nih.gov, last accessed on 19 October 2022) database. Multiple alignments of the ITS gene region were calculated using the software MUSCLE (default conditions for gap opening and gap extension penalties applied) and implemented in the software MEGA X version 10.2.4 (Molecular Evolutionary Genetics Analysis) [33]. The phylogenetic tree was calculated based on neighbor-joining (NJ) with bootstrap analysis including 1000 replicates.

### 4.4. Fungal Cultivation to Assess Secondary Metabolite Production

The fungal isolates were cultivated in Petri dishes containing PDA medium at room temperature for 7 to 10 days. The bioprocesses of fungal culture were performed as reported elsewhere [34] with minor modifications in triplicates. In short, the bioprocesses were carried out in an Erlenmeyer flask (150 mL) containing 50 mL of PDA broth (120 g/L potato, 10 g/L dextrose). About 1 × 10^4^ fungal spores/mL were inoculated into this medium and incubated under static conditions allowing fermentation for 15 days in the dark at room temperature (28 °C ± 2 °C).

### 4.5. Preparation of Extracts

Subsequently, the obtained filtrates were extracted in order of polarity using the solvents hexane (Hex) and ethyl acetate (AcOEt) (Synth, São Paulo, Brazil) (1:1:3) to obtain the metabolites. The resulting broth extracts were dried in a vacuum evaporator and the residues were resuspended with 10% dimethyl sulfoxide (DMSO). All fungal extracts were evaluated for their biological/antibacterial activities.

### 4.6. Assessment of Antimicrobial Activity of Fungal Metabolites on E. coli Strains Applying the Well Plate Method

The antibacterial potential of fungal metabolites against bacterial isolates was tested applying the well diffusion method [35] on Mueller-Hinton agar (MHA, Kasvi, Madrid, Spain) plates. In short, 100 µL volumes of an *E. coli* ATCC (25922) cell suspension and a cell suspension of the extended-spectrum beta-lactamase-(ESBL-) positive strain *E. coli* NCTC^®^ 13353 (Controllab, Rio de Janeiro, Brazil) with cell densities of 0.5 McFarland standard units each were inoculated on MH agar plates. Wells (6 mm) were loaded with 100 µL of extract each. Amoxillin at a concentration of 50 µg/mL was used as a positive control and 10% DMSO as a negative control. The plates were incubated for 24 h at 37 °C and the inhibition zone was recorded by measuring the diameter around the wells. Each experiment was performed in triplicates.

#### Minimum Inhibitory Concentration (MIC)

The minimum inhibitory concentration (MIC) was determined applying the modified microdilution method [36] with the same bacteria used for the diffusion approach described above. The fungal extracts were adjusted to an initial concentration of 3.2 mg/mL. The microdilution assay was performed in 96-well microtiter plates according to a previously described protocol. Briefly, fungal extracts were used in decreasing concentrations from 1.600 to 1.56 µg/mL. A volume of 10 µL bacterial inoculum was added with a final concentration of 10^5^ CFU/mL. Incubation was carried out for 16 h to 20 h at 37 °C. The assays were performed in duplicate and the reading was performed after the incubation period based on visual comparison with bacterial growth in the positive control well. The MIC value was defined as the lowest concentration, expressed in µg/mL, at which microorganisms did not show visible growth.

### 4.7. Cytotoxicity Bioassays

Cytotoxicity of the extracts was evaluated applying the Alamar Blue^TM^ assay [37] (Merck, Darmstadt, Germany) with MRC-5 (human fibroblast) cells grown in DMEM (Dulbecco’s Medium Eagle’s medium) supplemented with 10% fetal bovine serum kept in a CO_2_ incubator at 37 °C with an atmosphere containing 5% CO_2_. Cells were seeded in 96-well plates (0.5 × 10^4^ cells per well). After 24 h, extracts were dissolved in DMSO, added to each well (10 µg/mL) and incubated for 48 h. Doxorubicin (5 µg m/L) was used as a positive control. Negative controls (blanks) received the same amount of DMSO and had the same final DMSO concentrations as the samples (0.1%). Two hours before the end of the incubation, 10 µL of Alamar Blue^TM^ was added to each well. The resulting fluorescent signal was monitored with a multiplate reader at a range of 530–560 nm excitation wavelength and 590 nm emission wavelength. The fluorescent signals obtained with the assay were proportional to the number of vital cells in the sample.

### 4.8. Thin Layer Chromatography (TLC)

The selected extracts were dissolved in acetate (1 mg/mL) and applied with a fine-hole glass capillary tube. In detail, a volume of 10 µL of each fraction was applied over the entire length of the pre-mixed glass TLC plates coated with a silica gel 60 layer F254 (thickness 0.25 mm, Kieselgel 60 F254, Merck, Germany, size 20 × 20 cm). TLC chromatograms were obtained using optimized solvent systems (100% hexane, hexane/ethyl acetate 1:1—*v*/*v*, 100% ethyl acetate, ethyl acetate/methanol 1:1—*v*/*v*, 100% methanol) and examined under ultraviolet light at a wavelength of 365 nm. The metabolites were labeled.

### 4.9. Contact Bioautography

Contact bioautography was performed to determine the zone with antimicrobial activity as described [38] with minor modifications. The sterile materials obtained with the TLC approach described above were aseptically inoculated on the plates together with *E. coli* suspensions (MacFarland scale 0.5) and kept there for a period of 45 min in order to allow the metabolites to diffuse into the plates. Subsequently, the TLC platelets were removed from the surface of the plates with the help of sterile forceps and the plates were incubated in an inverted position for 48 h at 37 °C. Areas of inhibition were marked and the relevant Rf (retention factor) values were recorded and compared with the TLC chromatograms. All tests were performed in triplicates.

### 4.10. Liquid Chromatography-Mass Spectrometry (LC-MS/MS)

The crude extracts were analyzed with liquid chromatography coupled to mass spectrometry (Prominence UFLC, Shimadzu, Kyoto, Japan) and coupled to a mass spectrometer with quadrupole (Bruker Daltonics, Amazon Speed Ion Trap, Bremen, Germany). Five microliters of the dye samples were injected in C18 Shim-packs CLC-ODS(M)^®^ (250 mm x 4.6 mm, particle diameter 5 µm) using a binary phase (solvent A: water, solvent B: methanol) for the separations. Gradient elution was conducted at concentrations of 20–100% solvent B over 24 min at a flow rate of 1 mL/min. The obtained spectra were assessed based on visual interpretation of MS/MS spectral data.

## 5. Conclusions

The present study investigated the antimicrobial potential of metabolites of fungi isolated from a polluted stream in Manaus, Amazonas, Brazil. Five isolates (*A. stygium* WL1B5, *C. fructicola* WL3B9, *C. rosea* WL5B18, *C. rosea* WL8B28 and *T. harzianum* WL9B49) were associated with antimicrobial activity against *E. coli*. In the EtOAc extract of *A. stygium* WL1B5, two metabolites in particular were proposed: nodulisporone sodium and daidzein. These results confirmed that fungi isolated from polluted environments are promising sources of bioactive secondary metabolites, calling for further exploration as part of the search for new antibiotic drugs.

## Figures and Tables

**Table 1:** Mean values and standard deviation of the limnological parameters measured at the various sampling sites.

Limnological Parameters	Sampling Sites at Mindú Stream			
	Unit	Head Water	Central Point	Estuary
Temperature	°C	27 ± 0.1	27 ± 0.1	27 ± 0.1
pH	-	6.3 ± 0.1	7. 3 ± 0.2	6.1 ± 0.1
Dissolved oxygen (DO)	(Sat) %	3.2 ± 0.1	0.1 ± 0.1	0.1 ± 0.1
Chemical oxygen demand O_2_ (Conc.)	mgO_2_/L	57 ± 0.1	107 ± 0.2	151 ± 0.3
Turbidity	NTU	3.4 ± 0.2	3.9 ± 0.2	10 ± 0.3
Eletric conductivity	µScm^−1^	0.4 ± 0.1	47.6 ± 0.4	106.8 ± 0.1
Nitrate	mg/L	0.1 ± 0.1	0.2 ± 0.1	2 ± 0.1
Nitrite	mg/L	0.1 ± 0.1	0.1 ± 0.1	0.2 ± 0.1
Amonia	mg/L	0.1 ± 0.1	0.2 ± 0.0	1.2 ± 0.1
Total nitrogen	mg/L	1.4 ± 0.1	2.4 ± 0.6	8.7 ± 0.1
Total phosphorus	mg/L	0.1 ± 0.1	0.2 ± 0.2	0.4 ± 0.1
Orthophosphate	mg/L	0.1 ± 0.1	0.1 ± 0.1	0.3 ± 0.1

**Table 2:** Number of CFU (colony forming units)/mL, the total number of fungal isolates and the total number of isolates of aerobic fungi from three different sampling sites on the Mindú stream, Manaus, Amazonas. CFU = colony forming units.

Sample Site	CFU/mL	Total Isolates	Genera	Number of Isolates by Genus
Head water (03 01′07.31″ S 59 55′84″ W)	1.4 × 10	14	*Colletotrichum* sp. *Oidiodendrum* sp. *Mycelia sterilia*	1 1 12
Central point (03 04′28.4″ S 59 58′57.7″ W)	2.4 × 10	24	*Annulohypoxylon* sp. *Chloridium* sp. *Clonostachys* sp. *Oidiodendrum* sp. *Paecilomyces* sp. *Rodothorula* sp. *Trichoderma* sp. *Mycelia sterillia*	1 1 1 1 1 1 1 17
Estuary (03 06′58.4″ S 60 02′01.5″ W)	2.9 × 10	29	*Aspergillus* sp. *Clonostachys* sp. *Fusarium* sp. *Penicillium* sp. *Rodothorula* sp. *Trichoderma* sp. *Mycelia sterillia*	1 1 5 1 1 4 17

**Table 3:** Antibacterial activity of different solvent extracts based on hexane, EtOAc and crude extraction of fungal isolates from the Mindú stream assessed with *E. coli* ATCC 25922 and an ESBL-positive *E. coli* NCTC 13353.

Fungal Species	Zones of Inhibition (mm)					
	Bacterial Strains					
	*E. coli* ATCC 25922			*E. coli* NCTC 13353		
	Hex	EtOAc	CE	Hex	EtOAc	CE
*Annulohypoxylon stygium* WL1B5	-	35.9 ± 0.1	-	-	14 ± 1.4	5.95 ± 0.1
*Colletotrichum fructicola* WL3B9	7.9 ± 0.1	7.25 ± 0.4	6 mm	-	-	-
*Clonostachys rosea* WL5B18	-	19.8 ± 0.3	13.9 ± 0.1	-	4 ± 1.4	-
*Clonostachys rosea* WL8B28	1 ± 1.4	13.15 ± 1.2	26.95 ± 0.1	-	8 ± 1.4	18.9 ± 0.1
*Trichoderma harzianum* WL9B49	-	4.4 ± 0.8	-	-	-	-
Positive control (amoxillin 50 µg/mL)	20 mm			0 mm		
Negative control (10% DMSO)	0 mm			0 mm		

Hex—Hexane extract; EtOAc—Ethyl acetate extract; CE—Crude extract; extract volume 100 µL; incubation time and temperature 24 h/37 °C. The dash symbol (-) indicates no activity.

**Table 4:** Minimum inhibitory concentrations (MIC, µg/mL) of extracts of selected fungi with antimicrobial activity isolated from the Mindú stream.

Extract	Minimum Inhibitory Concentration of EtOAc Extracts (µg/mL)		
	Fungal Species and Strain Identity	Bacterial Strains	
		*E. coli* ATCC 25922	*E. coli* NCTC 13353
EtOAc	*Annulohypoxylon stygium* WL1B5	400 µg/mL	400 µg/mL
	*Clonostachys rosea* WL5B18	400 µg/mL	400 µg/mL
	*Clonostachys rosea* WL8B28	400 µg/mL	400 µg/mL
Positive control	amoxillin (12.5 µg/mL)		
Negative control	10% DMSO		

Initial extract concentration: 3.2 mg/mL; incubation time and temperature: 16/20 h/37 °C.

**Table 5:** TLC analysis and contact bioautography of mobile phases tested against *E. coli* ATCC 25922 with Rf values.

EtOAc Extract	Mobile Phase	Rf of Bands with Antimicrobial Activity
*A. stygium* WL1B5	Hex (100%)	0.15
	MeOH (100%)	0.86
*C. rosea* WL5B18	EtOAc/MeOH (1:1)	0.71
*C. rosea* WL8B28	EtOAc/MeOH (1:1)	0.78

Rf—retention factor; Hex—hexane; MeOH—methanol; EtOAc/MeOH—ethyl acetate/methanol.

## Data Availability

All relevant data are presented in the manuscript. Raw data of the study can be provided on reasonable request by the authors.
